# The *pbrB* Gene Encodes a Laccase Required for DHN-Melanin Synthesis in Conidia of *Talaromyces* (*Penicillium*) *marneffei*

**DOI:** 10.1371/journal.pone.0122728

**Published:** 2015-04-13

**Authors:** Ariya Sapmak, Kylie J. Boyce, Alex Andrianopoulos, Nongnuch Vanittanakom

**Affiliations:** 1 Department of Microbiology, Faculty of Medicine, Chiang Mai University, Chiang Mai, Thailand; 2 Department of Genetics, Faculty of Science, University of Melbourne, Victoria, Australia; Woosuk University, KOREA, REPUBLIC OF

## Abstract

*Talaromyces marneffei* (Basionym: *Penicillium marneffei*) is a significant opportunistic fungal pathogen in patients infected with human immunodeficiency virus in Southeast Asia. *T*. *marneffei* cells have been shown to become melanized *in vivo*. Melanins are pigment biopolymers which act as a non-specific protectant against various stressors and which play an important role during virulence in fungi. The synthesis of the two most commonly found melanins in fungi, the eumelanin DOPA-melanin and the allomelanin DHN-melanin, requires the action of laccase enzymes. The *T*. *marneffei* genome encodes a number of laccases and this study describes the characterization of one of these, *pbrB*, during growth and development. A strain carrying a PbrB-GFP fusion shows that *pbrB* is expressed at high levels during asexual development (conidiation) but not in cells growing vegetatively. The *pbrB* gene is required for the synthesis of DHN-melanin in conidia and when deleted results in brown pigmented conidia, in contrast to the green conidia of the wild type.

## Introduction

*Talaromyces marneffei* (Basionym: *Penicillium marneffei*) is an opportunistic human fungal pathogen endemic to Southeast Asia and Southern China [1–3]. Most cases of *T*. *marneffei* infection occur in immune-deficient hosts, especially those infected with human immunodeficiency virus (HIV), however, infections have also been reported in non-HIV children with the underlying immune defects (e.g. severe combined immunodeficiency (SCID), congenital lymphopenia, hyper-IgM syndrome, and hyper–IgE syndrome). Failure to treat infections is fatal, especially in children with primary immunodeficiency [2, 3]. *T*. *marneffei* is a dimorphic fungus with a thermally regulated dimorphic switch. As such, *T*. *marneffei* is capable of undergoing a transition from the saprophytic filamentous multicellular hyphae found in the environment (or *in vitro* at 25°C) to a unicellular yeast growth form *in vitro* at 37°C and during infection. During growth at 25°C, hyphae can also undergo asexual development (conidiation) to produce conidia, the infectious propagules. Hyphae differentiate by sequential production of an aerial stalk followed by the budding of the metula and phialide cell types from the stalk tip and culminating in the differentiation of conidia, by budding from phialides, to produce long chains of uninucleate asexual spores [4, 5]. Conidia exhibit a green coloration. In related fungal species, conidial coloration is due to the synthesis of DHN-melanin (1,8-dihydroxynaphthalene melanin) produced from polyketides made from acetate precursors [6].

Melanins are hydrophobic pigment biopolymers with a negative charge formed by oxidative polymerization of phenolic or indolic compounds. Generally, melanins present in brown to black colors, however, other colors such as green are found. Melanins act as non-specific shields against various stressors (e.g. UV, oxidizing agents, enzymatic lysis, and extreme high and low temperatures) and play an important role during virulence in fungi [6–8]. In plant pathogenic fungi, melanins at the appressoria aid in the penetration of plant tissues [9]. In human pathogenic fungi, melanin protects cells from the harsh environment within the host, including shielding the cells from components of the immune response and antifungal agents [8, 9]. There are three classes of melanins synthesized in fungi; eumelanins, allomelanins, and pheomelanins. The eumelanin DOPA-melanin (3,4-dihydroxyphenylalanine melanin) and the allomelanin DHN-melanin (1,8-dihydroxynaphthalene melanin) are the two most common melanins in fungi and the best characterized. Both DOPA-melanin and DHN-melanin production requires the action of laccases. Laccase (p-diphenol:dioxygenoxidoreductase, EC 1.10.3.2) is a member of the multicopper oxidase (MCO) family of enzymes. The relationship amongst MCO members correlates with the type of enzyme, its function, and the source organism [10–13]. Laccases can catalyze the oxidation of a wide variety of organic (especially aromatic) and inorganic compounds. Laccases oxidize DOPA to dopaquinone that can be used to produce DOPA-melanin [6, 7, 9]. In *Cryptococcus neoformans*, *lac1* encodes a laccase that when deleted leads to mutants being unable to synthesize the brown colored DOPA-melanin and this results in reduced virulence in a mouse infection model [14]. Laccases are also involved in generating the pigmentation of conidia. This pigmentation, which can be observed as a variety of colors, is attributed to DHN-melanin synthesis and requires the activity of a laccase to polymerize 1,8-DHN to form the DHN-melanin polymer [6, 15]. Deletions of the laccases encoded by the *Aspergillus nidulans yA* and *Aspergillus fumigatus abr2* genes result in a lack of the green colored DHN-melanin in conidia [16–18]. *A*. *nidulans yA* mutants exhibit yellow conidia, whereas *A*. *fumigatus abr2* mutants produce brown conidia [16, 18, 19].

Melanization in *T*. *marneffei* has been identified both *in vitro* and during infection. *T*. *marneffei* is capable of synthesizing both DHN- and DOPA-melanin [20–22]. The polyketide synthase WA is responsible for DHN-melanin synthesis during asexual development [22]. Laccase activity has been detected in extracts from yeast cells [20] and *T*. *marneffei* yeast cells can produce DOPA-melanin using L-DOPA supplemented in culture medium [21]. Both DOPA- and DHN-melanin biosynthetic pathways require the function of laccases [6, 15]. However, the specific laccase(s) required have not been identified and the enzymatic activities necessary for melanin biosynthetic steps have not been verified in detail. To examine the type of melanin occurs during conidiation, the DHN-melanin synthesis inhibitor tricyclazole [23–25] was added to the culture medium of *T*. *marneffei* grown at 28°C. It was found that the addition of tricyclazole altered the green coloration of conidia suggesting that the melanin synthesized during *T*. *marneffei* conidiation is a DHN-melanin. The genome of the *T*. *marneffei* type strain harbors 10 genes that encode multicopper oxidase proteins. Phylogenetic analysis showed that *T*. *marneffei pbrB* gene encodes a laccase that shares a common evolutionary origin with fungal laccases functioning in DHN-melanin synthesis. To test this hypothesis we deleted the *pbrB* gene and characterized its function during growth and development. By analysing the gene expression pattern and protein localization we show that the *pbrB* gene is required for the synthesis of DHN-melanin in conidia.

## Materials and Methods

### Strains and culture conditions

All transformants were produced from a uracil auxotrophic strain (G526, Δ*pkuA pyrG* ^*-*^) derived from *Talaromyces marneffei* FRR2161 (CBS 334.59, ATCC18224) [26, 27]. Strain G526 lacks the *pkuA* gene encoding the ku70 protein which functions in non-homologous DNA end joining repair [28]. Therefore, genetic transformation is mediated via homologous recombination only. G526 harbors a spontaneous, loss-of-function mutation in *pyrG* (orotidine-5’-phosphate decarboxylase encoding gene) selected by growth in the presence of 5-fluoroorotic acid [27]. All *pyr*G^+^ transformants and the *T*. *marneffei* G681 (Δ*pkuA*::*pyrG* ^*+*^) strain used as a control, were maintained on ANM medium containing 1% (w/v) glucose, 10 mM ammonium sulfate [26]. The *T*. *marneffei* F4 strain isolated from an AIDS patient (CBS 119456) [29] was maintained on malt extract (ME) agar. Conidial suspensions of transformants and the G526 strain were harvested from conidiating colonies growing on ANM solid medium at 28°C for 1 week. Colonies on petri dishes were flooded with sterilized phosphate buffer saline, gently scraped, filtered through Miracloth (Calbiochem), and recovered by centrifugation.

### Inhibition of reductase enzyme function assay

Conidia were harvested from *T*. *marneffei* F4 colonies cultured on ME agar at 28°C for 1 week. A suspension of 10^6^ conidia was spread on ME agar with and without 30 μg/ml tricyclazole in 6-well plate. The plate was incubated at 28°C for 1 week.

### RT-PCR

The expression of *pbrB* was investigated by RT-PCR. RNA samples were extracted from *T*. *marneffei* F4 cultured in brain heart infusion (BHI) broth at 28°C and 37°C for 3 days. RNA was extracted and used as a template to produce cDNA using Omniscript reverse transcription kit (Qiagen). The *pbrB* cDNA was then amplified using specific primers PmLac25-F and L25Re. The 18s rRNA was amplified as a control using Pm1 and Pm2 primers [30]. Primers used in this study are listed in Table 1.

**Table 1:** List of primers used in this study.

Primer name	Sequence (5’ > 3’)
PmLac25-F	GTGCTGAATCAAGGTGACGA
L25Re	CATTGCATCCATGTACTCG
Pm1	ATGGGCCTTTCTTTCTGGG
Pm2	GCGGGTCATCATAGAAACC
PmLac25U	GCCAGCCTGATTGCAGCG
PmLac25UL	GTTCAGCACGCTCGGTCG
iLL25EC1	CTTGATATCGAACGTTAGGGTGGGCGCCCCAAATG
iUL25EC1	CCTAACGTTCGATATCAAGTTCAAAATGACACG
fpEGFPC1	TTGATATCGTGAGCAAGGGCGAGGAG
rpEGFPC1	AAATCGATTCTAGATCCGGTGGATCCCG
PmLac4F	CGCACAGCGTTATTCAGT
PmLac4R	CACTACATGCGGCGAGGT

### Construction of transformation vectors

To construct the Δ*pbrB* plasmid, PmLac25U and PmLac25L primers were used to amplify the *pbrB* gene containing approximately 1.4 kb of upstream and downstream untranslated regions (UTRs) from *T*. *marneffei* strain G681. Purified PCR product (4,747 bp) was ligated into pGEM-T Easy (Promega) to produce the *pbrB* plasmid (plac25). This plasmid was digested with *Bgl*II and *Stu*I to remove the *pbrB* coding region (138 bp before ATG to 155 bp before stop codon). The *Bgl*II/*Stu*I *pbrB* plasmid was ligated to a *Bam*HI/*Eco*RV fragment containing the *pyrG* blaster selectable marker cassette (pAB4342 [27]) to generate pplilac25. The linearized insert from pplilac25 was excised by *Not*I and purified before transformation. For the complementation construct, p25N9, the *NotI* fragment from plac25 was ligated into a *Not*I–digested/dephosphorylated pAB4342 vector.

In order to generate the *pbrB*(p)::*pbrB*::*GFP* strain, plac25 was amplified by inverse PCR using iLL25EC1/iUL25EC1 primers. The PCR product was digested with *Eco*RV and *Acl*I and then ligated with *Eco*RV/*Cla*I fragment containing *egfp* to produce plac25GFP. The *egfp* coding region was inserted at the 5’ end of *pbrB*, 87 bases after the start codon. The *egfp* coding region was amplified from the pEGFP-C1 vector (kindly provided by Dr. Amornrat O’Brien [31]) using fpEGFPC1/rpEGFPC1 primers. The whole *pbrB*(p)::*pbrB*::*GFP*fragment was excised from plac25GFP by *Not*I digestion and cloned into *Not*I—digested/dephosphorelated pAB4342 to generate pGFPLac4N.

### Generation of transformants

*T*. *marneffei* strain G526 was used to generate the Δ*pbrB*::*pyrG* ^*+*^ mutant and *pbrB*(p)::*pbrB*::*GFP* strain. DNA-mediated transformation was performed using PEG-mediated transformation protocol described previously [27]. The PyrG^*+*^ transformants were selected on medium without uracil. To generate a Δ*pbrB pyrG* ^*-*^ strain, the Δ*pbrB*::*pyrG* ^*+*^ strain was inoculated onto ANM agar containing 1 mg/ml 5-fluoroorotic acid to select for loss of the *pyrG* cassette via homologous recombination between the inverted CAT repeats that flank *pyrG* in the construct [32]. To generate the Δ*pbrB pbrB* ^*+*^ complementation strain, the Δ*pbrB pyrG* ^*-*^ strain was transformed with p25N9 and PyrG^*+*^ transformants were selected.

Genetically modified *T*. *marneffei* transformants were examined by PCR and/or Southern blot analysis [26]. The *pbrB* amplification was performed using PmLac25F/L25Re primers, while fpEGFPC1/L25Re primers were for *egfp-pbrB* amplification. The *T*. *marneffei fetC* gene (PMAA_057450) was amplified as an internal control of PCR using PmLac4F/PmLac4R primers. The hybridization probe for Southern blot analysis was the *pbrB* gene amplified by PCR using PmLac25U/PmLac25L primers.

### Germination and growth rate assays

For germination assays, conidia (10^6^) of each strain were inoculated in a six well microtitre plate containing BHI broth and the plate was incubated at 28°C or 37°C for 24 hours. Aliquots of the broth culture were placed on slides to count germinating conidia. Three replicates were performed counting at least 100 cells each time. For growth rate assays, approximately 10 conidia were spread onto 3 plates of ANM agar and incubated at 28°C for 3 days. The diameter of 5 individual colonies was measured daily over a period of 7 days. Mean and standard error of the mean (SEM) values were calculated and analyzed for statistical significance using GraphPad software (http://www.graphpad.com/).

### Fluorescence microscopy

Conidia (10^6^) of the *pbrB*(p)::*pbrB*::*GFP* strain were inoculated into two flasks containing 50 ml of BHI broth. Each flask was incubated in a shaking incubator at 28°C or 37°C for 3 days. Mycelia from the 28°C culture were poured onto Miracloth and rinsed with cold PBS before microscopic examination. Fungal cells from the 37°C culture were harvested by centrifugation, washed with cold PBS, placed on a microscope slide and examined using an Olympus Provis AX70 fluorescence microscope.

To examine conidiating fungal cells, a piece of ANM agar block (about 10 x 10 x 5 mm) was placed on a sterile glass slide. Conidia from the *pbrB*(p)::*pbrB*::*GFP* strain were inoculated on each side of agar block. A cover glass was placed on the top of the agar block. The slide culture was incubated in a humidified chamber at 28°C for 10 days. The agar block was removed and fungal cells that remained attached to the slide were fixed with absolute ethanol before air drying in a biosafety cabinet. Fungal cells on the slide were incubated with 5 mg/ml *Trichoderma harzianum* cell wall lysing enzyme (Sigma-Aldrich, UK) dissolved in osmotic buffer [27] at 37°C for 30 minutes and then the solution was poured off. Fungal cells were permeabilized by the addition of 0.2% Triton X-100 in PBS for 5 minutes. Slides were washed with cold PBS solution. Immunofluorescence staining was performed as previously described [20]. Briefly, slide cultures were blocked with Superblock Blocking Buffer (Pierce, USA) at 37°C for 2 hours. The slides were incubated with 10 μg/ml of anti-melanin IgM antibody, kindly provided by Dr. Sirida Youngchim [20], at 37°C for 1.5 hours. Slides were washed with PBS and then incubated with a dilution 1:100 of rhodamine-labeled goat anti-mouse IgM antibody (Jackson Immunoresearch Laboratories, USA) at 37°C for 1.5 hours. After washing, cells were observed under a fluorescence microscope. The negative control samples were produced by omitting the anti-melanin IgM binding step in order to assess the background caused by non-specific binding of secondary antibody. The Superblock Blocking buffer used to dilute anti-melanin IgM was added, instead of using anti-melanin IgM, before incubating with secondary antibody.

To examine chitin deposition, calcofluor white staining was performed as previously described [33]. Three-day-old fungal cells were harvested from BHI broth culture as described above and the cells were washed with PBS before staining.

## Results

### The green pigment synthesized during conidiation in *T*. *marneffei* is dihydroxynaphthalene melanin

**Fig 1:**
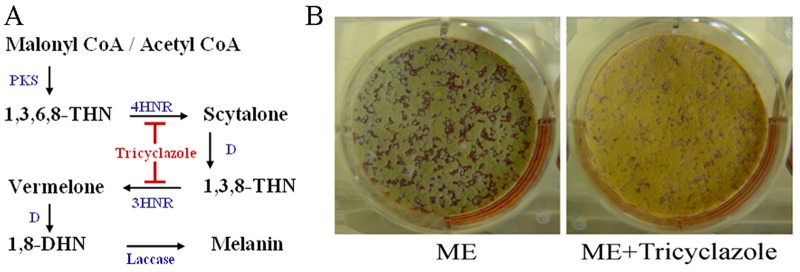
The effect of tricyclazole on melanization during asexual development. (A) The generic DHN melanin biosynthetic pathway [15, 24, 43]. Enzymes acting at each step include polyketide synthase (PKS), tetrahydroxynaphthalene reductase (4HNR), trihydroxynaphthalene reductase (3HNR), dehydratase (D), and laccase. Intermediate metabolites including 1,3,6,8-tetrahydroxynaphthalene (1,3,6,8-THN), scytalone, 1,3,8-trihydroxynaphthalene (1,3,8-THN), vermelone, and 1,8-dihydroxynaphthalene (1,8-DHN). (B) At 28°C, *T*. *marneffei* F4 colonies appear green as a result of the pigmented conidia. The addition of 30 μg/ml tricyclazole, which inhibits two reductases (4HNR and 3HNR) functioning during DHN melanin synthesis, results in yellow conidiation.

To examine if the green coloration of conidia was attributed to DHN-melanin, *T*. *marneffei* conidia were inoculated onto medium with or without the inhibitor tricyclazole. Tricyclazole inhibits the two hydroxynaphthalene reductases functioning in DHN-melanin synthetic pathway (Fig 1A) [23–25]. In the absence of tricyclazole, *T*. *marneffei* conidia appeared green (Fig 1B). In contrast, in a presence of tricyclazole, the *T*. *marneffei* conidia appeared yellow (Fig 1B). This suggests that the synthesis of DHN-melanin contributes to the green coloration of conidia during asexual development.

### The *T*. *marneffei* genome encodes ten multicopper oxidases

**Fig 2:**
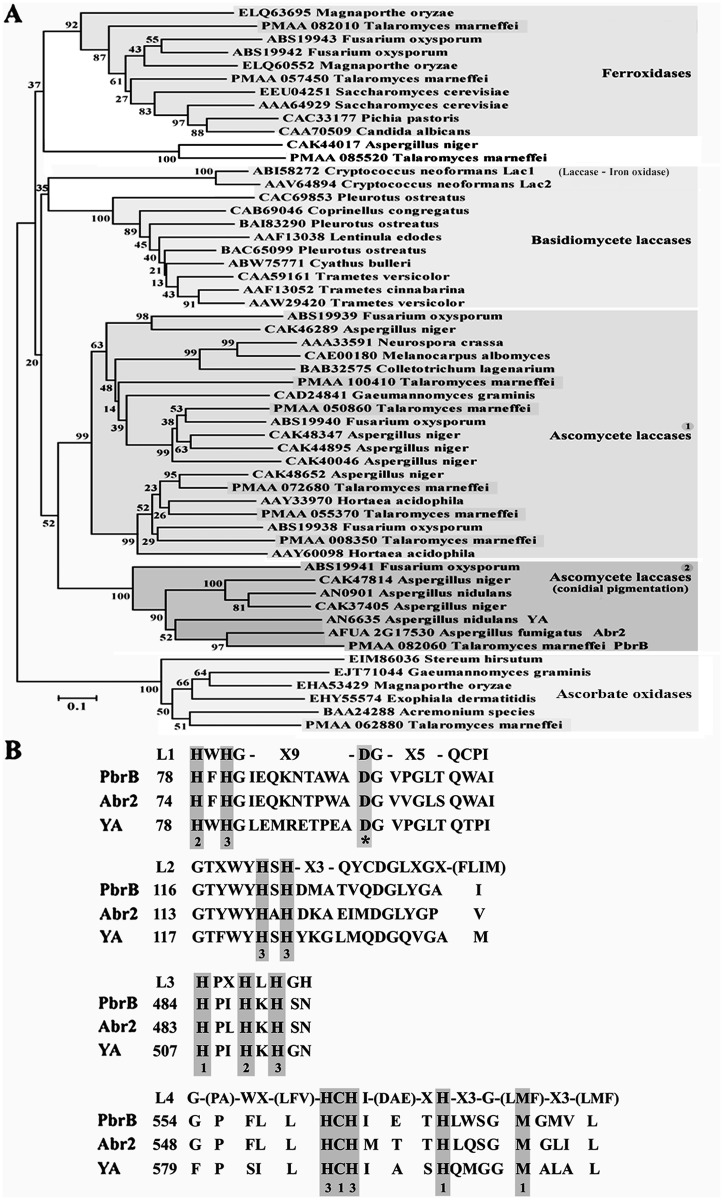
Phylogenetic analysis and partial sequence alignment. (A) Putative fungal MCO sequences from a number of species, including those predicted in *T*. *marneffei*, were obtained from Genbank (http://www.ncbi.nlm.nih.gov/protein/) and used to build sequence alignments in CLUSTALW. The alignment was then used to construct a relatedness tree using MEGA 6 software. Phylogenetic relationships of the 55 MCOs were inferred using the Neighbor-Joining method and bootstrap tested (1000 replicates). Branch lengths of the tree are drawn to scale and bootstrap support indicating at the branch sites. Fungal MCOs that share a common ancestry with more than 60% bootstrap value are shaded in gray. Functions are defined at each clade based on characterized function of MCO members. (B) A partial alignment representing the conserved motifs of copper binding sites and L1–L4 signature sequences found in *T*. *marneffei* PbrB (PMAA_082060) and its orthologs *A*. *fumigatus* Abr2 (AFUA_2G17530) and *A*. *nidulans* YA (AN6635). The numbers in front of each sequence indicate the amino acid position of a particular protein. Cu binding motifs are identified above the sequences, numbers including 1, 2, and 3 are type 1 Cu, type 2 Cu, and type 3 Cu. The asterisk indicates the potential proton donor for the reaction intermediates.

Laccases are members of the multicopper oxidase (MCO) family, which also includes ferroxidases and ascorbate oxidases. To identify laccase encoding genes in *T*. *marneffei* the *C*. *neoformans* Lac1 and *A*. *fumigatus* Abr2 (Fig 2A) encoding genes were used as a query sequence in blast searches of the *T*. *marneffei* ATCC 18224 genome sequence (GenBank, NCBI). This identified two highly homologous genes (PMAA_072680 and PMAA_085520) (*Cnlac1* homology of 88% and 78%, respectively) that were then also used for additional blast searches to retrieve related sequences in the *T*. *marneffei* genome. These homology searches identified 10 genes encoding putative multicopper oxidases (PMAA_008350, PMAA_050860, PMAA_055370, PMAA_057450, PMAA_062880, PMAA_072680, PMAA_082010, PMAA_082060, PMAA_085520, and PMAA_100410). Generally, laccases contain four copper atoms including type1 Cu, type 2 Cu, and a pair of type 3 Cu. The patterns of conserved amino acids coordinated with each type of copper are HXHG, HXH, HCHXXXHXXXM/F/L, and HXXHXH, which occupy the L1–L4 signature sequences starting from the N-terminus [34–36]. We found that 9 of the *T*. *marneffei* MCOs (excluding PMAA_062880) have these conserved patterns (see S1 Fig). Previous phylogenetic studies reveal that MCO sequences are often clustered with other genes according to function, fungal division, and source organism [10, 11, 13]. To predict which *T*. *marneffei* MCO participates in conidial DHN-melanin synthesis, we combined 55 fungal MCO sequences and performed alignments using CLUSTALW (http://www.genome.jp/tools/clustalw/). Tree construction was conducted in MEGA version 6 [37] using the Neighbor-Joining method. The 55 fungal MCO sequences were separated into 5 clades with well-supported branches (>90% bootstrap support) (Fig 2A). Each clade was highlighted and named according to sequences with known functions and fungal divisions. The ascomycete laccase lineage can be divided into 2 clades supported with very high bootstrap values (99% and 100%). Focusing on the second clade containing *T*. *marneffei* PbrB, this clade comprises of characterized laccases functioning in conidial DHN-melanin synthesis. *T*. *marneffei* PbrB is more closely related to *A*. *fumigatus* Abr2 than *A*. *nidulans* YA [16–18].

### *pbrB* is expressed during asexual development and localizes to all cell types of the conidiophore

**Fig 3:**
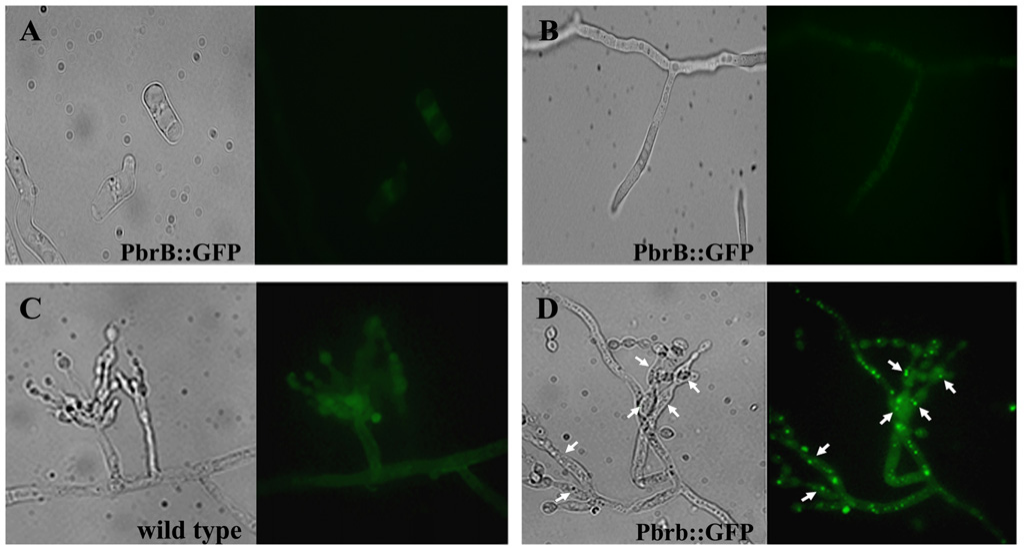
Expression and localization of PbrB in *T*. *marneffei*. Epifluorescence microscopy of the *T*. *marneffei* strain expressing the PbrB::GFP fusion in vegetative cells grown at 37°C (A) or 28°C (B) for 3 days. No GFP fluorescence was noted in either cell type. Epifluorescence microscopy of the *T*. *marneffei* conidiophores from the wild type (G681) strain (C) and the strain expressing the PbrB::GFP fusion (D) under bright field and fluorescence optics. The PbrB::GFP fusion is observed as distinct spots (white arrows) in metulae and phialides. Strains were grown on slides at 28°C for 10 days. Microscopic images were captured at 1000X magnification.

The expression of *pbrB* was investigated by RT-PCR. RNA samples were extracted from *T*. *marneffei* F4 grown in BHI broth at 28°C and 37°C for 3 days. A transcript was not detected under these conditions suggesting there is little or no *pbrB* expression in vegetative hyphal or yeast cells (data not shown). To analyze *pbrB* expression further, a strain was generated that expresses a fusion construct in which *pbrB*, expressed from the native promoter, is fused to GFP (*pbrB*(p)::*pbrB*::*GFP*). Conidia of the *pbrB*(p)::*pbrB*::*GFP* strain were inoculated into BHI broth and cultured at 28°C and 37°C for 3 days. In support of the RT PCR analysis, GFP fluorescence was not detected in vegetative hyphal cells at 28°C or vegetative yeast cells at 37°C under standard conditions (Fig 3A and 3B). In addition, vegetative cells were grown at 28°C and 37°C in a variety of other types of medium (ANM, Synthetic Dextrose and Malt Extract) and in the presence of copper with low glucose (0.2%) or under acidic condition pH 5.0, which are known laccase inducing conditions [38]. No signal from PbrB::GFP could be detected under any of these conditions after 3, 5 and 7 days of incubation (data not shown).

A slide culture of the *pbrB*(p)::*pbrB*::*GFP* strain was prepared to observe expression of *pbrB* during conidiation. Compared to the negative control (Fig 3C), strong GFP fluorescence was observed in conidiophores of the *pbrB*(p)::*pbrB*::*GFP* strain, suggesting that PbrB is expressed during asexual development (Fig 3D). The PbrB::GFP protein was localized as distinct spots in metulae and phialides (Fig 3D).

**Fig 4:**
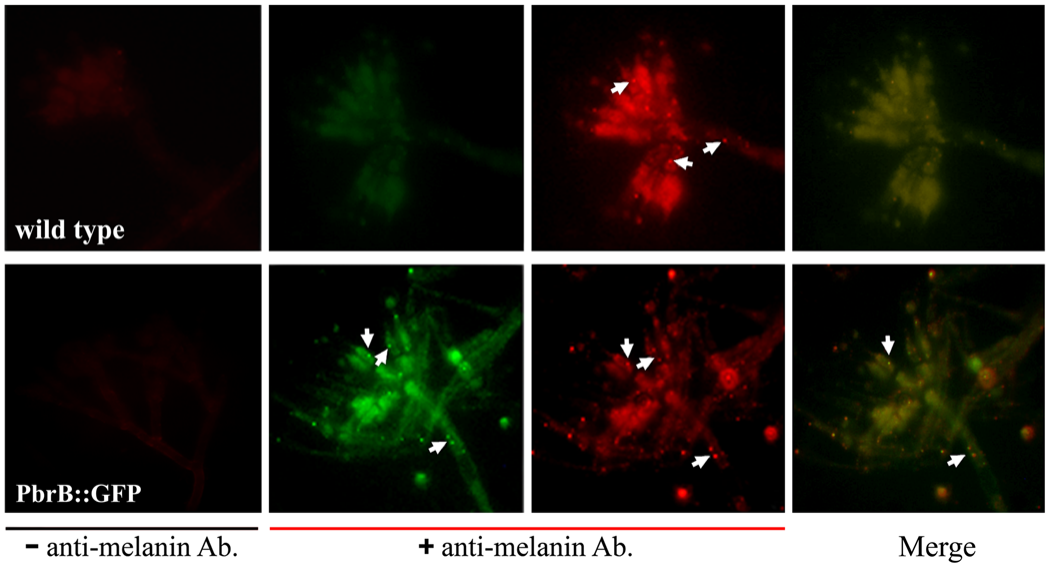
Localization of PbrB and melanin during conidiation in *T*. *marneffei*. Epifluorescence microscopy after immunostaining using an anti-melanin antibody staining for the wild type control (G681) and the PbrB::GFP fusion strain after growth on slides at 28°C for 10 days. To assess non-specific staining by the rhodamine-labeled secondary antibody (goat anti-mouse IgM antibody), samples were processed without the primary anti-melanin antibody step (labeled as—anti-melanin Ab.). Red fluorescent signals of melanin or melanin-like particles are observed in both wild type and PbrB::GFP fusion strain (+ anti-melanin Ab.). Green fluorescent signals indicate sites of PbrB::GFP proteins. The merged image shows co-localization of PbrB::GFP and melanin-labeled fluorescence. White arrows identify sites of fluorescence signals which melanin-labeled particles co-localize with PbrB::GFP proteins. Microscopic images were captured at 1000X magnification.

To investigate if PbrB co-localizes with melanin, melanin was detected in the *pbrB*(p)::*pbrB*::*GFP* strained by immunolabeling with an anti-melanin antibody. Immunostaining detected strong fluorescence in phialide cells with some weaker staining in the stalk, metulae and conidial cell types. There was also some punctate melanin staining in the various cell types. This staining was highly co-localised with the fluorescence form the PbrB::GFP fusion protein (Fig 4). This supports the hypothesis that PbrB is likely to be involved in melanin biosynthesis during conidiation.

### *T*. *marneffei pbrB* is required for the melanization of conidia

**Fig 5:**
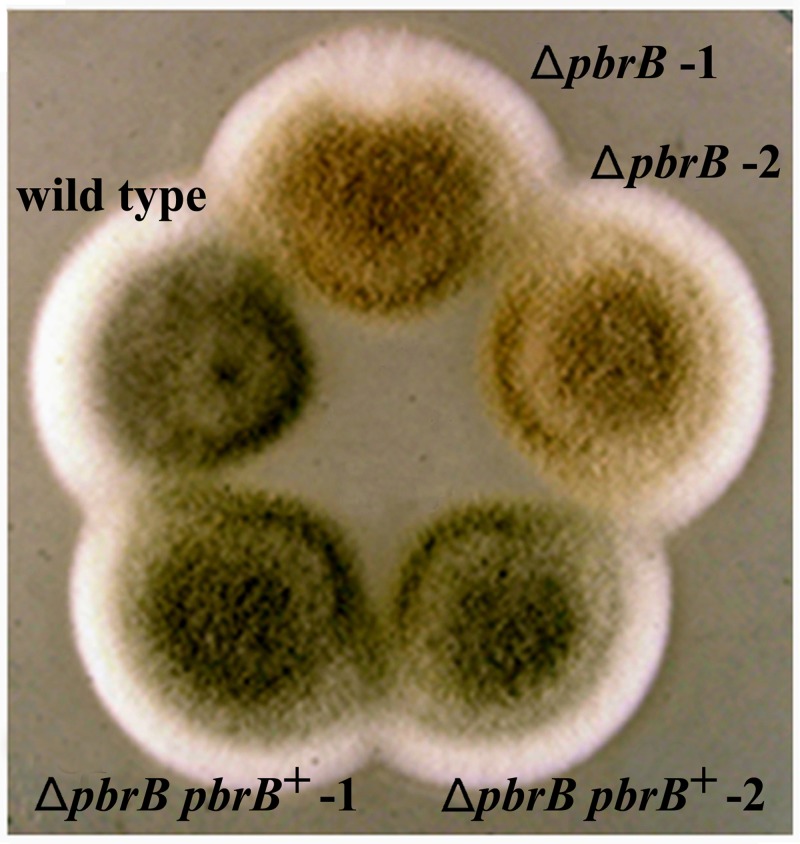
Macroscopic morphologies of the wild type and mutant *T*. *marneffei* strains. Colonies of the wild type *T*. *marneffei* control strain G681, Δ*pbrB* 1&2 strains, and Δ*pbrB pbrB* ^*+*^ 1&2 complemented strains grow on ANM agar at 28°C for 14 days. Conidiation of the Δ*pbrB* strains is brown compared to the green conidiation of the parental and complemented strains.

**Fig 6:**
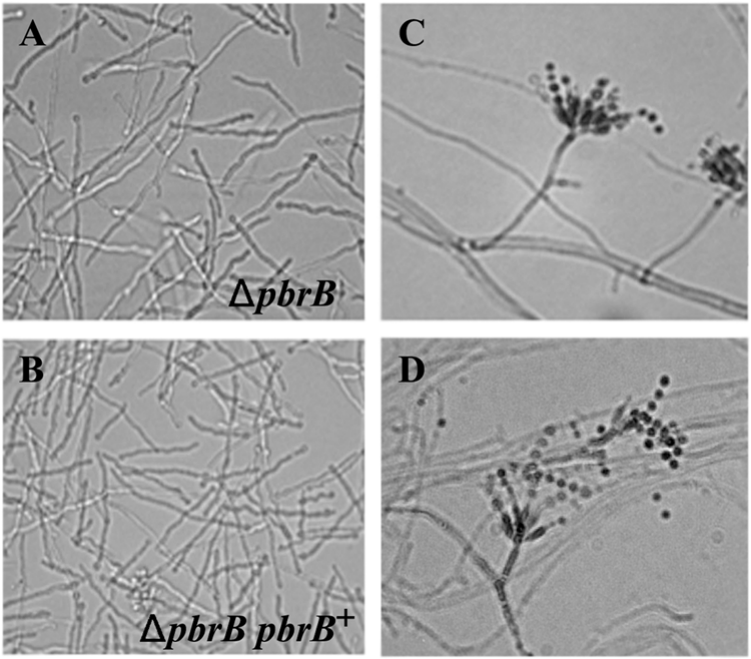
Germination and growth of the Δ*pbrB* strain. Microscopic imaging of the *T*. *marneffei* Δ*pbrB* and Δ*pbrB pbrB* ^+^ strains grown at 28°C for 24 h to assess germination (A, B) and 7 days to observe conidiophores (C, D). Microscopic images were captured at 200X (A, B) and 400X (C, D) magnification.

To characterize the role of *pbrB* in *T*. *marneffei*, Δ*pbrB* mutants were generated. Two independent mutants were characterized with respect to conidiation, germination and growth. In order to examine morphogenesis during conidiation, Δ*pbrB* conidia were grown on ANM plates. In contrast to the wild type control, which exhibits green colored conidiation at 28°C after 14 days, conidiation of the Δ*pbrB* mutants appeared a light brown color (Fig 5). Reintroduction of a wild type copy of *pbrB* at the native locus (Materials and Methods) in the Δ*pbrB* mutant complemented the conidiation phenotype (Fig 5). Slide cultures of the Δ*pbrB* and Δ*pbrB pbrB* ^*+*^ complemented strains growing on ANM agar at 28°C for 7 days were prepared to examine the morphology of conidiophores. Deletion of *pbrB* did not result in any morphological defects in any of the conidiophore cell types (Fig 6C and 6D). The change in phenotype indicates that PbrB has a unique function that cannot be compensated by other *T*. *marneffei* laccases.

To assess if deletion of *pbrB* and the consequent change in pigmentation of the conidia affected germination, 10^6^conidia of the Δ*pbrB* and Δ*pbrB pbrB* ^*+*^ strains were inoculated in BHI broth and incubated at 28°C or 37°C for 24 hours then the number of germinated conidia were counted. There was no difference in the germination of the Δ*pbrB* and Δ*pbrB pbrB* ^*+*^ strains at 28°C (Fig 6A and 6B) or 37°C (data not shown). Hyphal cells from the Δ*pbrB* and Δ*pbrB pbrB* ^+^ strains were grown at 28°C for 3 days and examined microscopically. Staining of hyphae with 1 μg/μl calcofluor white showed no defects in chitin deposition nor was any morphological difference detected (data not shown).

**Fig 7:**
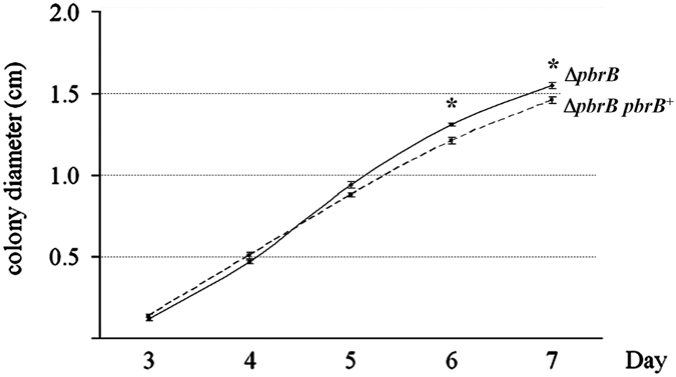
Vegetative growth rate effects in the Δ*pbrB* mutant. Radial growth rates were assessed by measuring colony diameters for the Δ*pbrB* and Δ*pbrB pbrB* ^+^ strains growing at 28°C on ANM medium. The line plots the growth rates of Δ*pbrB* (solid line) and Δ*pbrB pbrB* ^+^ (dashed line). Error bars present standard error of the mean (SEM). Asterisks indicate significantly difference between strains determined by p-values (p = 0.002 and 0.015). X axis is time and Y axis is the mean colony diameter.

Growth rates of the Δ*pbrB* and Δ*pbrB pbrB* ^+^ strains were determined by measuring colony diameters daily over the course of 7 days on ANM medium at 28°C. Mean and standard error of the mean (SEM) were calculated and statistical differences were assessed using the t-test. The differences in growth rates between Δ*pbrB* and Δ*pbrB pbrB* ^+^ strains at 6 and 7 days were found to be statistical significant (p = 0.002 and 0.015, respectively) (Fig 7).

### Loss of *T*. *marneffei pbrB* does not affect sensitivity to stressor*s*

Deletion of *pbrB* results in brown conidiation color, in contrast to the green coloration of wild type. This suggests that like *A*. *fumigatus* and *A*. *nidulans*, *pbrB* in *T*. *marneffei* is required for the synthesis of DHN-melanin. As melanins protect fungal cells against environmental assaults, the susceptibility of the Δ*pbrB* mutant to a variety of stresses was determined. Ten-fold dilutions of Δ*pbrB* and Δ*pbrB pbrB* ^*+*^ conidial suspensions were dropped on ANM and BHI agar containing 2% H_2_O_2_ (oxidative stress), 20 μg/ml SDS (cell wall stress), 3 μM Congo Red (cell wall stress), 1 M sorbitol (osmotic stress), 0.6 M NaCl (salt stress), 5 mM NaNO_2_ (nitrosative stress) or antifungals (0.15 μg/ml amphotericin B, 0.1 μg/ml clotrimazole, 40 μg/ml fluconazole, and 0.04 μg/ml itraconazole) and incubated at either 28°C and 37°C for 7 days. In addition, susceptibility to antifungal activity of macrophage (J774) was examined. However, no differences in stress susceptibility were observed (data not shown).

Cytoplasmic protein extracts from the wild type and Δ*pbrB* mutant cultured in brain heart infusion broth at 37°C for 3 days were capable of catalyzing L-DOPA (data not shown). In addition, Δ*pbrB* cells growing in BHI medium at 37°C for 5 days showed equivalent staining as the wild type using the anti-melanin antibody. These results suggest that melanization of vegetative cell types is not dependent on *pbrB* and this is consistent with the results from the susceptibility tests using the various stress agents.

## Discussion

The multicopper oxidase (MCO) family comprises enzymes that typically contain four copper atoms classified into three types (type 1 Cu, type 2 Cu and type 3 Cu). Members of MCO family include laccases, ferroxidases, ascorbate oxidases, bilirubin oxidases, CueO, and ceruloplasmin [10, 11, 34, 35]. Among MCO members, laccases can be found in various organisms (e.g. plants, fungi, bacteria and insects), but not in humans. A large number of laccases are produced in many basidiomycete and ascomycete fungal species and a variety of physiological roles have been reported including morphogenesis, stress defense, fungal pathogen/host interaction and delignification [7, 12, 36]. In pathogenic fungi, laccases have attracted attention due to their involvement in melanization and the correlation with host invasion and protection against host immunity. Laccases contribute to the melanization pathways producing the most commonly isolated fungal melanins; DOPA- and DHN-melanin [7, 9]. Generally, melanins are deposited in the cell wall of conidia and fungal cells [6, 15]. This study has determined the role of a developmentally regulated laccase, encoded by *pbrB*, during DHN-melanin synthesis in conidia. Neither the *pbrB* transcript nor the GFP-tagged protein in a *pbrB*(p)::*pbrB*::*GFP* strain could be detected during vegetative hyphal or yeast growth. However, GFP fluorescence was observed in conidiophores of the *pbrB*(p)::*pbrB*::*GFP* strain, suggesting that *pbrB* is only expressed during asexual development in *T*. *marneffei*. Likewise in *A*. *fumigatus* and *A*. *nidulans*, expression of the orthologous *abr2* and *yA* genes characteristically occurs during conidiophore development but not during vegetative growth [16, 18]. Phylogenetic examination showed that *T*. *marneffei* PbrB is more closed to *A*. *fumigatus* Abr2 than *A*. *nidulans* YA (Fig 2A). Moreover, *T*. *marneffei* Δ*pbrB* colonies displayed brown-pigmented conidia that resemble those of the Δ*abr2* strain [17, 18]. Our data suggests that *T*. *marneffei* PbrB is polymerizing polymers of 1,8-DHN to form DHN-melanin, as has been shown in *A*. *fumigatus* [39]. *T*. *marneffei pbrB* is located within a genomic cluster of genes with homology to those required for conidial pigment biosynthesis in other systems and includes PMAA_082010 (conidial biosynthesis oxidase AbrA), PMAA_082020 (conidial biosynthesis protein AygA), PMAA_082030 (1,3,6,8—tetrahydroxynaphthalene reductase ArpA), PMAA_082040 (conidial pigment biosynthesis scytalone dehydrogenase ArpA) and PMAA_082120 (conidial pigment polyketide synthase AlbA) (see S2A Fig). This cluster is conserved in *A*. *fumigatus* (S2B Fig).

**Fig 8:**
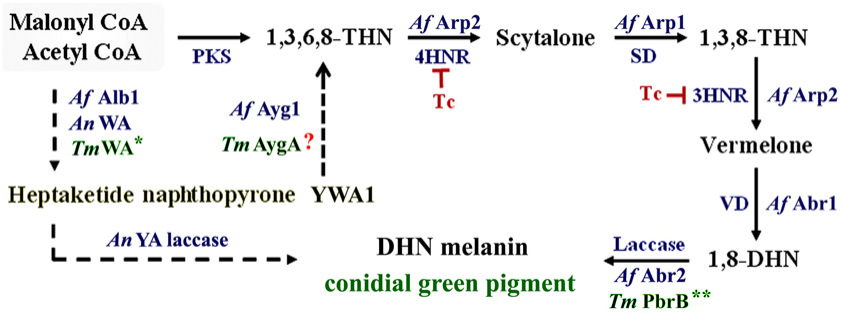
Predicted DHN melanin synthesis pathway in *T*. *marneffei*. A consensus biochemical pathway for DHN melanin synthesis based on what is known from other fungal systems is shown [15, 39, 50]. Processing steps of the well-known DHN pathway are presented with dark arrows. PKS, polyketide synthase; 4HNR, tetrahydroxynaphthalene reductase; SD, scytalone dehydratase; 3HNR, trihydroxynaphthalene reductase; VD, vermelone dehydratase; 1,3,6,8-THN, tetrahydroxynaphthalene; 1,3,8-THN, trihydroxynapthalene; 1,8-DHN, dihydroxynaphthalene. Tc, tricyclazole, can inhibit both reductases (4HNR and 3HNR) presented in a model pathway. Distinctive steps described in *A*. *nidulans* (*An*), *A*. *fumigatus* (*Af*), *T*. *marneffei* (*Tm*) are shown with dashed arrows. Asterisks, * and **, refer to data from previous study [22] and this study, respectively.

The biochemical pathway for DHN-melanin production was first described by Wheeler and Bell (1988) and has been well characterized both biochemically and genetically in many ascomycetes. The genus *Aspergillus* comprises many species and pigmented conidia appear in various colors among species. Effects of inhibitors on DHN-melanin synthesis (using tricyclazole and phthalide) and DOPA-melanin synthesis (using kojic acid and tropolone) in a range of *Aspergillus* species demonstrate that differences in the amounts and types of pigments and melanins synthesized by related species are likely to be a common theme [40, 41]. Among *Aspergillus* and *Penicillium* species, most exhibit green to bluish green conidial pigments and the use of inhibitors of melanization pathways has revealed that most of these pigments are made from pentaketide metabolites [41]. The starting carbon units are acetate [25, 42], malonyl CoA and/or acetyl CoA [6, 15, 43] and are catalyzed by a polyketide synthase (PKS) in the first step before subsequent processing through enzymatic steps to produce DHN-melanin [6, 9, 12, 23]. Mutation of the gene encoding the PKS in *A*. *nidulans (wA*), *A*. *fumigatus* (*pksP*) and *T*. *marneffei* (*wA*) similarly results in white, non-melanized conidia [44, 45, 22]. While this early part of the pathway seems to be conserved amongst these fungi, differing results have been shown when tests are conducted with the DHN-melanin synthesis inhibitor tricyclazole. Tricyclazole specifically affects the reductases that reduce 1,3,6,8-tetrahydroxynaphthalene to scytalone and 1,3,8-trihydroxynaphthalene to vermelone [23–25, 46]. Tricyclazole alters the conidial color phenotype of *A*. *fumigatus* but it does not affect conidial coloration of *A*. *nidulans*, *A*. *flavus*, and *A*. *parasiticus* [41, 47]. Further studies showed that the conidial pigment biosynthesis in *A*. *fumigatus* and *A*. *nidulans* possess some steps that are distinct from the general model of DHN-melanin biosynthetic pathway (Fig 8) [6, 15]. Instead of producing tetrahydroxynaphthalene (THN) from the starter units, *A*. *nidulans* and *A*. *fumigatus* utilize malonyl CoA and Acetyl CoA to produce heptaketide naphthopyrone YWA1 by heptaketide synthase (HKS) [44, 48]. Although YWA1 is a precursor for green conidial pigmentation in both *A*. *nidulans* and *A*. *fumigatus*, the downstream metabolic steps are different in the two organisms. In *A*. *nidulans*, YA laccase catalyzes YWA1 to produce the green pigment of conidia. In contrast, *A*. *fumigatus* YWA1 is catalyzed through a hydrolytic polyketide-based shortening step to produce 1,3,6,8-THN by Ayg1 [49]. A homolog of *A*. *fumigatus ayg1* is also presents in *T*. *marneffei* genome (see S2 Fig). As the addition of tricyclazole affected conidial color of *T*. *marneffei*, it suggests that hydroxynaphthalene reductases function in DHN-melanin synthetic pathway (Fig 8). Similar to *A*. *fumigatus*, YWA1 should be converted to 1,3,6,8-THN in *T*. *marneffei*. Then 1,3,6,8-THN is processed through additional steps to produce 1,8-DHN. Finally, 1,8-DHN is polymerized by PbrB laccase to form DHN-melanin, which appears as the green color of *T*. *marneffei* conidia (Fig 8).

Melanization of *T*. *marneffei* has been described previously, however the type of melanin was not determined [20]. In this study, we found that *pbrB* encodes a laccase enzyme expressed during conidiation. PbrB is required for the synthesis of DHN-melanin in conidia and when deleted results in brown conidiation, in contrast to the green conidiation of wild type. The existence of additional uncharacterized multicopper oxidase-encoding genes in the *T*. *marneffei* genome suggests that in addition to DHN-melanin, *T*. *marneffei* may also have the capacity to produce DOPA-melanin depending on growth conditions and supply of precursors.

## Supporting Information

S1 Fig Patterns of copper binding sites in *T*. *marneffei* MCOs. Sequence alignments showing the general copper signature sequences (L1–L4) found in fungal laccases [34, 35]. Site of the first amino acid of each sequence is indicated next to PMAA number. The copper binding residues are highlighted and note the type of copper (1, 2, and 3). Asterisk is a potential proton donor. Type 1 copper ligand (M/L/F) superscripted with I/II/II refers to redox potential class from low to high [35].
(TIF)

S2 Fig Schematic of the putative gene cluster involved in conidial pigmentation. (A) Genomic region in *T*. *marneffei* (Tm) from gene PMAA_082000 to PMAA_08120, which encompasses a cluster of genes, predicted to be required for conidial pigment synthesis. Genes required for conidial pigment biosynthesis are colored brown (PMAA_082010 oxidase, *abrA*), blue (PMAA_082020 *aygA*), orange (PMAA_082030 1,3,6,8-tetrahydroxynaphthalene reductase, *arpB*), aqua (PMAA_082040 scytalone dehydratase, *arpA*), red (PMAA_080260 laccase, *pbrB*) and green (PMAA_080260 polyketide synthase, *wA*). (B) *A*. *fumigatus* gene cluster involved in DHN melanin synthesis. This gene cluster is conserved in *A*. *fumigatus* (Af) (AFUG_2G17530, *abr2;* AFUG_2G17540, *abr1*; AFUG_2G17550 *ayg1*; AFUG_2G17560 *arp2*; AFUG_2G17580 *arp1* and AFUG_2G17600 *alb1*).
(TIF)
