# Supplementary figures and images for: The pbrB Gene Encodes a Laccase Required for DHN-Melanin Synthesis in Conidia of Talaromyces (Penicillium) marneffei
Source: PLoS One. 2015 Apr 13;10(4):e0122728. doi: 10.1371/journal.pone.0122728 (PMC4395095; doi:10.1371/journal.pone.0122728)

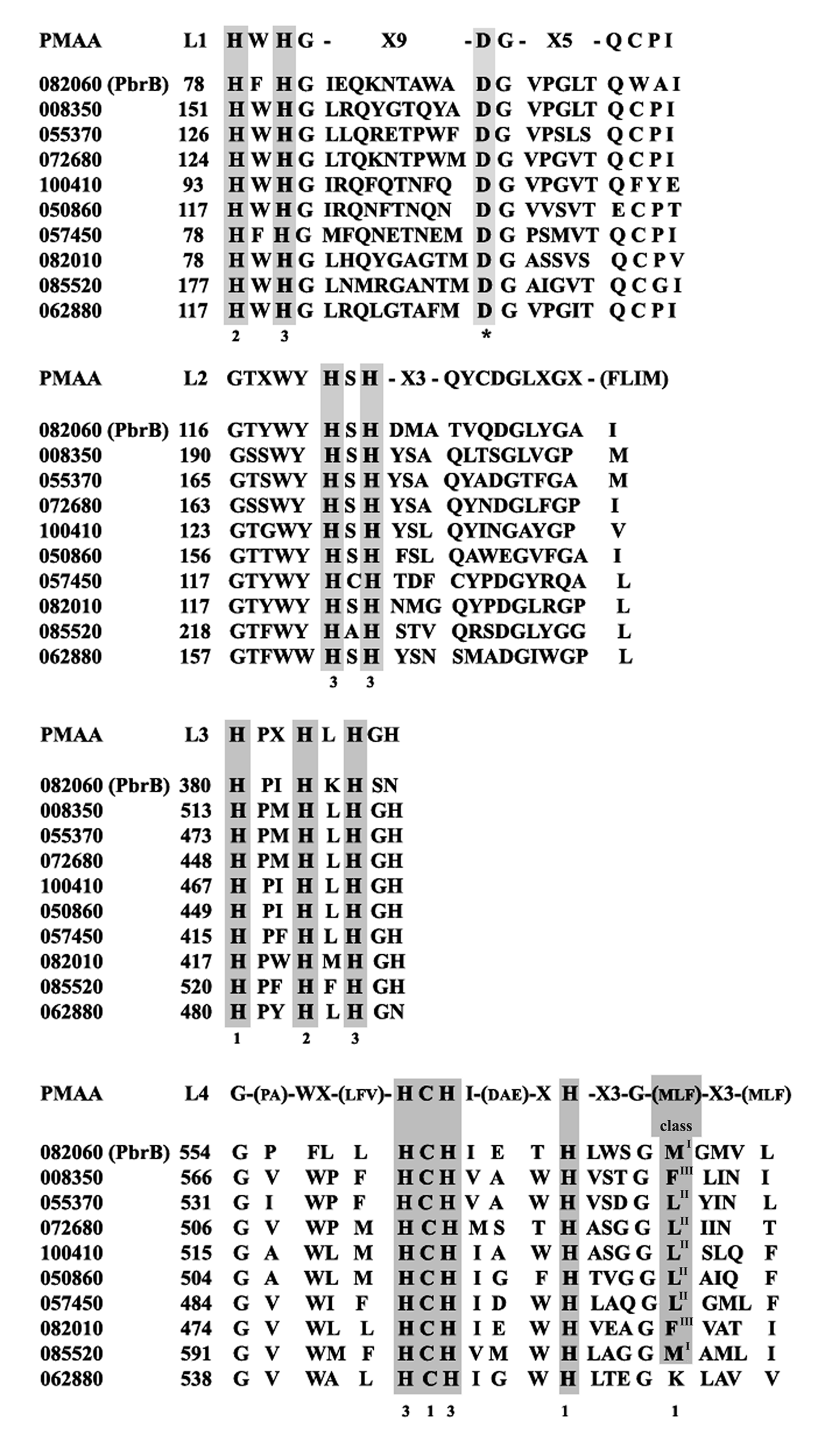

Supplement: S1 Fig — Sequence alignments showing the general copper signature sequences (L1–L4) found in fungal laccases [34, 35]. Site of the first amino acid of each sequence is indicated next to PMAA number. The copper binding residues are highlighted and note the type of copper (1, 2, and 3). Asterisk is a potential proton donor. Type 1 copper ligand (M/L/F) superscripted with I/II/II refers to redox potential class from low to high [35]. (TIF) [file pone.0122728.s001.tif]

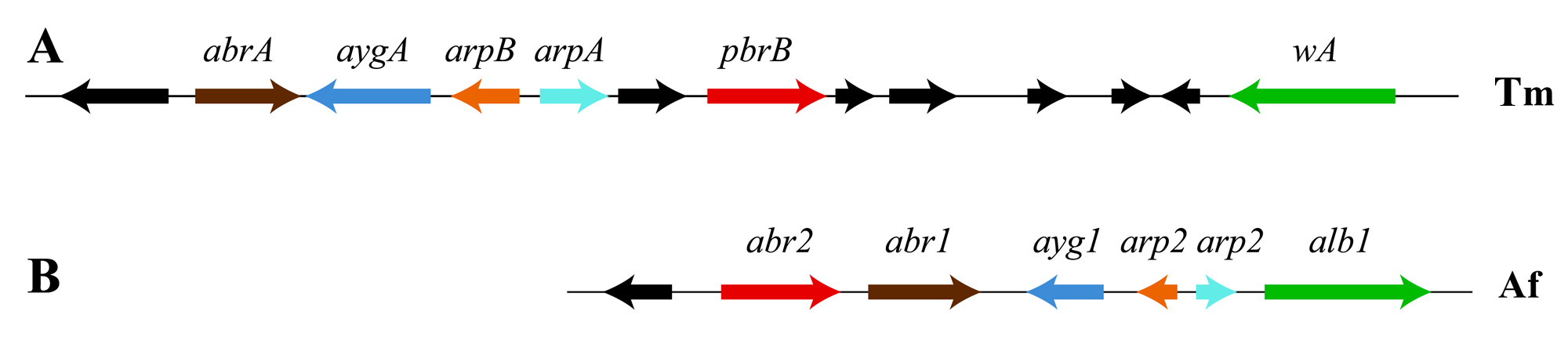

Supplement: S2 Fig — (A) Genomic region in T. marneffei (Tm) from gene PMAA_082000 to PMAA_08120, which encompasses a cluster of genes, predicted to be required for conidial pigment synthesis. Genes required for conidial pigment biosynthesis are colored brown (PMAA_082010 oxidase, abrA), blue (PMAA_082020 aygA), orange (PMAA_082030 1,3,6,8-tetrahydroxynaphthalene reductase, arpB), aqua (PMAA_082040 scytalone dehydratase, arpA), red (PMAA_080260 laccase, pbrB) and green (PMAA_080260 polyketide synthase, wA). (B) A. fumigatus gene cluster involved in DHN melanin synthesis. This gene cluster is conserved in A. fumigatus (Af) (AFUG_2G17530, abr2; AFUG_2G17540, abr1; AFUG_2G17550 ayg1; AFUG_2G17560 arp2; AFUG_2G17580 arp1 and AFUG_2G17600 alb1). (TIF) [file pone.0122728.s002.tif]
